# The “Connectivome Theory”: A New Model to Understand Autism Spectrum Disorders

**DOI:** 10.3389/fpsyt.2021.794516

**Published:** 2022-02-07

**Authors:** Leonardo Zoccante, Marco Luigi Ciceri, Luigi Alberto Gozzi, Gianfranco Di Gennaro, Nicoletta Zerman

**Affiliations:** ^1^Child and Adolescent Neuropsychiatry Unit, Maternal-Child Integrated Care Department, Integrated University Hospital Verona, Verona, Italy; ^2^Autism Spectrum Disorders Regional Centre of Verona, Verona, Italy; ^3^Department of Pathology and Diagnostics, Integrated University Hospital Verona, Verona, Italy; ^4^Department of Surgery, Dentistry, Paediatrics and Gynaecology, University of Verona, Verona, Italy

**Keywords:** autism(ASD), connective tissue, microglia, connectome, medical hypothesis, development, psyche and soma, new model

## Abstract

The classical approach to autism spectrum disorders (ASD) is often limited to considering their neuro-functional aspects. However, recent scientific literature has shown that ASDs also affect many body systems and apparatuses such as the immune system, the sensory-motor system, and the gut-brain axis. The connective tissue, a common thread linking all these structures, may have a pathogenetic role in the multisystem involvement of ASD. Depending on its different anatomical sites, the connective tissue performs functions of connection and support; furthermore, it acts as a barrier between the external and internal environments, regulating the interchange between the two and performing immunological surveillance. The connective tissue shares a close relationship with the central nervous system, the musculoskeletal system and the immune system. Alterations in brain connectivity are common to various developmental disorders, including ASD, and for this reason here we put forward the hypothesis that alterations in the physiological activity of microglia could be implicated in the pathogenesis of ASD. Also, muscle hypotonia is likely to clinically correlate with an altered sensoriality and, in fact, discomfort or early muscle fatigue are often reported in ASDs. Furthermore, patients with ASD often suffer from intestinal dysfunctions, malabsorption and leaky gut syndrome, all phenomena that may be linked to reduced intestinal connectivity. In addition, at the cutaneous and subcutaneous levels, ASDs show a greater predisposition to inflammatory events due to the lack of adequate release of anti-inflammatory mediators. Alveolar-capillary dysfunctions have also been observed in ASD, most frequently interstitial inflammations, immune-mediated forms of allergic asthma, and bronchial hyper-reactivity. Therefore, in autism, altered connectivity can result in phenomena of altered sensitivity to environmental stimuli. The following interpretative model, that we define as the “connectivome theory,” considers the alterations in connective elements of common mesodermal origin located in the various organs and apparatuses and entails the evaluation and interpretation of ASDs through also highlighting somatic elements. We believe that this broader approach could be helpful for a more accurate analysis, as it is able to enrich clinical evaluation and define more multidisciplinary and personalized interventions.

## Introduction

Autism spectrum disorder (ASD) is a complex condition characterized by the involvement of multiple organs and systems. The Diagnostic and Statistical Manual of Mental Disorders (DSM) 5th edition defines the autism spectrum as a wide group of heterogeneous neurodevelopmental disorders, characterized on a symptomatological level by social interaction anomalies through both verbal and non-verbal communication impairments, as well as by patterns of repetitive and stereotyped interests and behaviors (1). Recent epidemiological studies in the United States reported an ASD prevalence in up to 1 in every 54 8-year-old children (2). Historically, ASD has been described mainly as a neuro-functional alteration. Nevertheless, an increasing body of evidence is showing that, in addition to the known neurobehavioral characteristics, structural, and tissue alterations have been observed on several levels (3). The aim of this opinion paper is to describe how connective tissue could represent the “*grand unificateur*” of the human body, as suggested in a paper by A.I. Kapandji published in 2012, and how it could be involved in the development of ASD symptomatology (4).

## Connective Tissue

**Figure 1:**
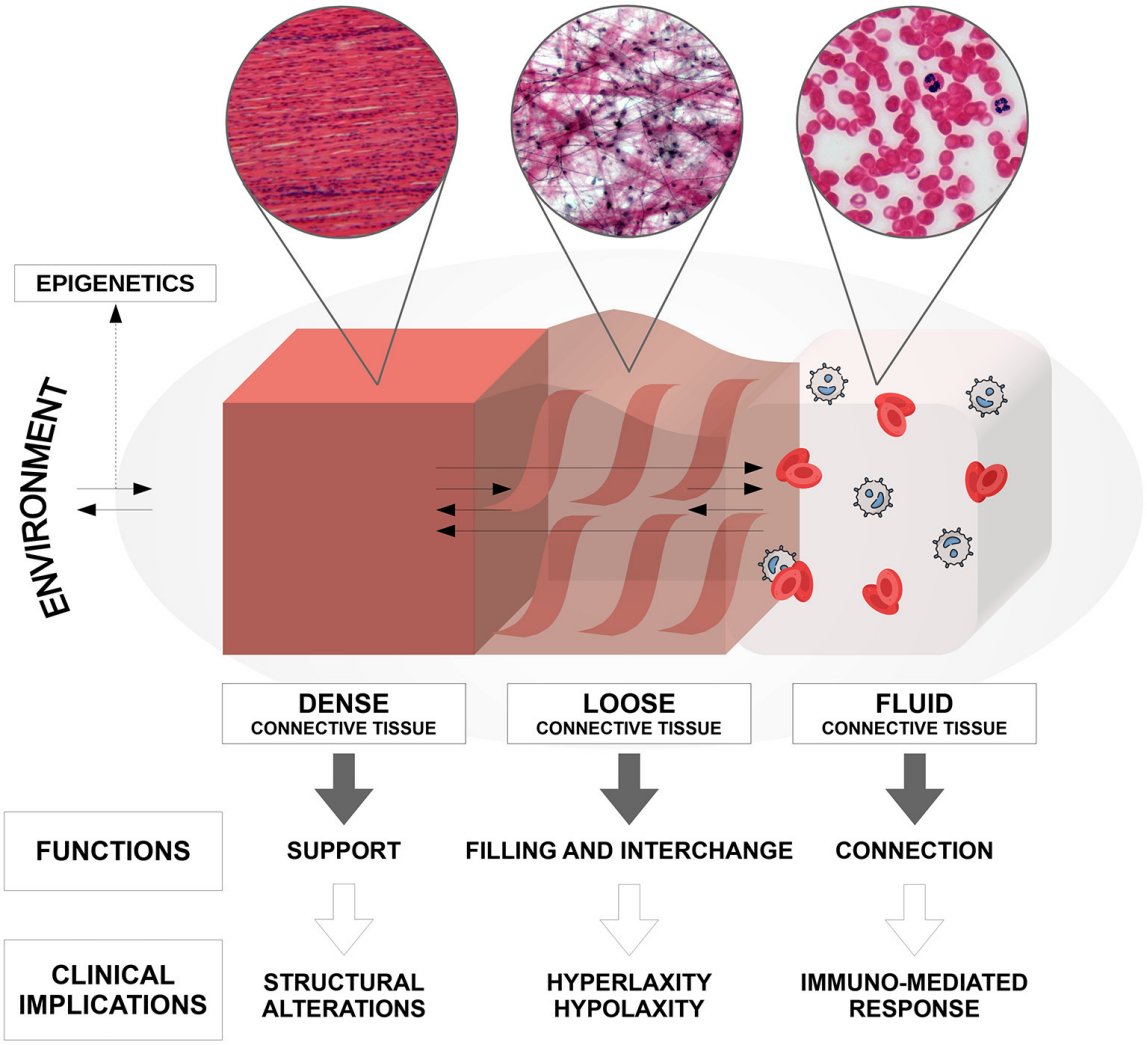
Composition, anatomo-structural functions, and clinical implications of the connective tissue in the human body.

Connective tissue, which mainly originates from the mesodermal embryonic germ layer, is present in all body systems and organs. Until now, only structural and adhesion roles have been attributed to connective tissue. Currently it is believed to perform, depending on the component of its matrix (dense, loose, or fluid), all of the following functions (Figure 1): connection, regulation, nourishment, immunological regulation, shaping, protection of the organism, and reserve (adipose tissue) (5). In addition, recent studies have shown that connective tissue also plays an important role in the communication between different body sites, besides vascular blood and lymphatic perfusion (6). In fact, new data suggests the existence of the so-called *interstitium*, a structural “cavity” found in interstitial spaces within the connective tissue that allows the transmission of long-distance signals, although its actual presence is still a matter of debate. Furthermore, it has been recognized that this *interstitium* has a shock-absorbing function to protect the organs, which does not follow the traditional vascular-nerve communication routes (7). Alterations in connective tissue at different levels are commonly observed within the autism spectrum and this allows us to understand more precisely the somatic manifestations of the disorder, which are not often taken into consideration in common diagnostic practice. In fact, many studies report connective tissue alterations in specific body sites, but no one has yet explained how these alterations are distributed throughout the entire body in subjects affected by ASD (4, 8).

### Brain and Connectome

Functions similar to those of connective tissue can also be found in the brain's white matter, especially when considering microglia, cells of mesodermal origin. In fact, white matter fibers play a peculiar connecting role, particularly in promoting the arborization of axonal connections and in enabling coordination of immunological pathways. Microglia constitutes between 10 and 15% of white matter (9) and performs different functions during the developmental stages of the human body. Most notably, it contributes to neurogenesis, axonal sprouting, brain wiring, and synaptic pruning. These macrophage-like myeloid cells are also implicated in a wide array of processes such as inflammation modulation, defense against infections, and the elimination of dead cells (10).

It is clear that alterations in microglia functions during developmental phases are associated with faulty synaptic maturation and profound alterations of the connectome, that is the totality of neuronal connections on both structural and functional levels (11). Evidence of an anomalous connectome, especially when compared to a healthy sample, has been obtained through functional magnetic resonance imaging studies that attributed this phenomenon to microglial activation in relation to oxidative and toxic-inflammatory stress of neuronal fibers (myelinated, large diameter, and long range fibers) (12). During the life path, the transition of microglia from a homeostatic surveillance state to an activated state has been observed in response to infection or damage. In the activated state, microglia facilitates antimicrobial surveillance or tissue repair programs, thus promoting the restoration of homeostasis (13–15). Microglial activation, reported by several authors as a neurobiological marker of impaired central connectivity, has been documented in post mortem neuropathological studies. Furthermore, glial cell dysfunctions have been linked to behavioral disturbances present in various developmental neuropsychiatric conditions, including ASD (16). Moreover, beyond the role played in brain development and homeostasis, recent genetic studies have suggested that microglia contributes to the pathogenesis of neurodevelopmental disorders, depending on the type of cerebral maturation and in relation to states of greater or lesser activation (10, 17). Furthermore, the establishment of an archive of radiological data such as the one created by the Autism Brain Imaging Data Exchange (ABIDE) Project has confirmed, on the one hand, the presence of short-range hyper-connections in the parietal site and in the sub-cortical fibers and, on the other, signs of hypo-connection of cortico-cortical connecting transcallosal fibers. Most authors also concord with results suggesting volumetric anomalies and network alterations in the different neurobiological systems in ASD (18, 19).

In brain tissue, communication between internal and external environments occurs through exchange activity with pericytes and other cells that form the blood-brain barrier. Therefore, microglial activity is also influenced by factors originating outside the central nervous system (CNS). The complex interplay between genetics and the environment is critical for proper brain development. During this phase, epigenetic regulation allows the brain to adapt to the specific environment in which the individual is growing, even during the prenatal stage. Recent evidence suggests that microglia plays an important role in responding to pre- and postnatal environmental stimuli through the epigenetic interface, modifying gene expression and thus acting as an effector of synaptic plasticity (20). A sophisticated crosstalk between the enteral nervous system and intestinal microbiome in the gut appears to be particularly critical for various facets of CNS physiology, including the development of microglia and its functions (21). Indeed, several neurodevelopmental disorders and psychiatric conditions, including ASD, have the presence of synaptic defects in common, therefore suggesting that alterations in the physiological activity of microglia could be implicated in their pathogenesis (22).

### Musculoskeletal System

Hypermobility has quite a high prevalence in ASDs, to the point that authors tend to include autism among hypermobility spectrum disorders (HSD) (20).

Joint, muscle and tendon structures are arranged on a single kinetic chain aimed at creating a precise movement. The mobility of a tendon or joint depends mainly on their elastin content, tropocollagen, or fibrillin fibers and these anatomical sites tend to become more rigid from childhood to adulthood to allow the development of muscle strength (23). Consequently, the more a muscle develops strength, the more it loses its elasticity. This progressive decrease in mobility can be found especially in the dominant side of the body and in males (23).

Although there are clinical tests capable of identifying the degree of muscle involvement, it is not always possible to distinguish hypotonia (muscle) from laxity (tendon) or hypermobility (joint); and, in autism, it is particularly difficult to determine the prevalence of one condition over the others (18).

Different and non-homogeneous connective tissue textures are present inside bone, depending on the function. Bone-densitometry studies show that, depending on the affected body area, bones present different connective tissue densities—compact and rigid textures where they must support body weight and structure (e.g., lumbar or pelvic vertebrae) and more rarefied textures where the body does not need the same load support (fingers, for instance) (19, 20).

ASD patients with significant degrees of hypermobility frequently report fatigue, sensory alterations, and coordination disorders (24, 25). Hypermobility is either ascribable to muscle hypotonia, characterized by excessive tendon elasticity, or to a greater degree of mobility than expected in relation to age (18). The clinical presence of hypotonic muscles is correlated with altered proprioceptive sensoriality, which interferes with motor sequence planning, starting from the actual location of the body segments in space. Muscle discomfort or early muscle fatigue can in turn be related to the need for greater muscle contraction to perform the movement (23, 26).

Teeth are fixed in the alveolar bone through the connective structures of the periodontal ligament. In people with autism, connective tissue alterations, in particular the prevalence of elastic fibers, can imply greater laxity which causes diastasis and dental malposition due to bad habits, atypical swallowing or lingual thrust (27). Anxiety traits typically observed in autism can be the cause of the wearing down of dental enamel due to nocturnal bruxism (28–30). The reduced gingival seal, associated with increased connective laxity and the aforementioned alterations in the oral cavity, results in poor general oral hygiene often found in autistic subjects (31, 32).

### Connective Tissue Interfaces

**Figure 2:**
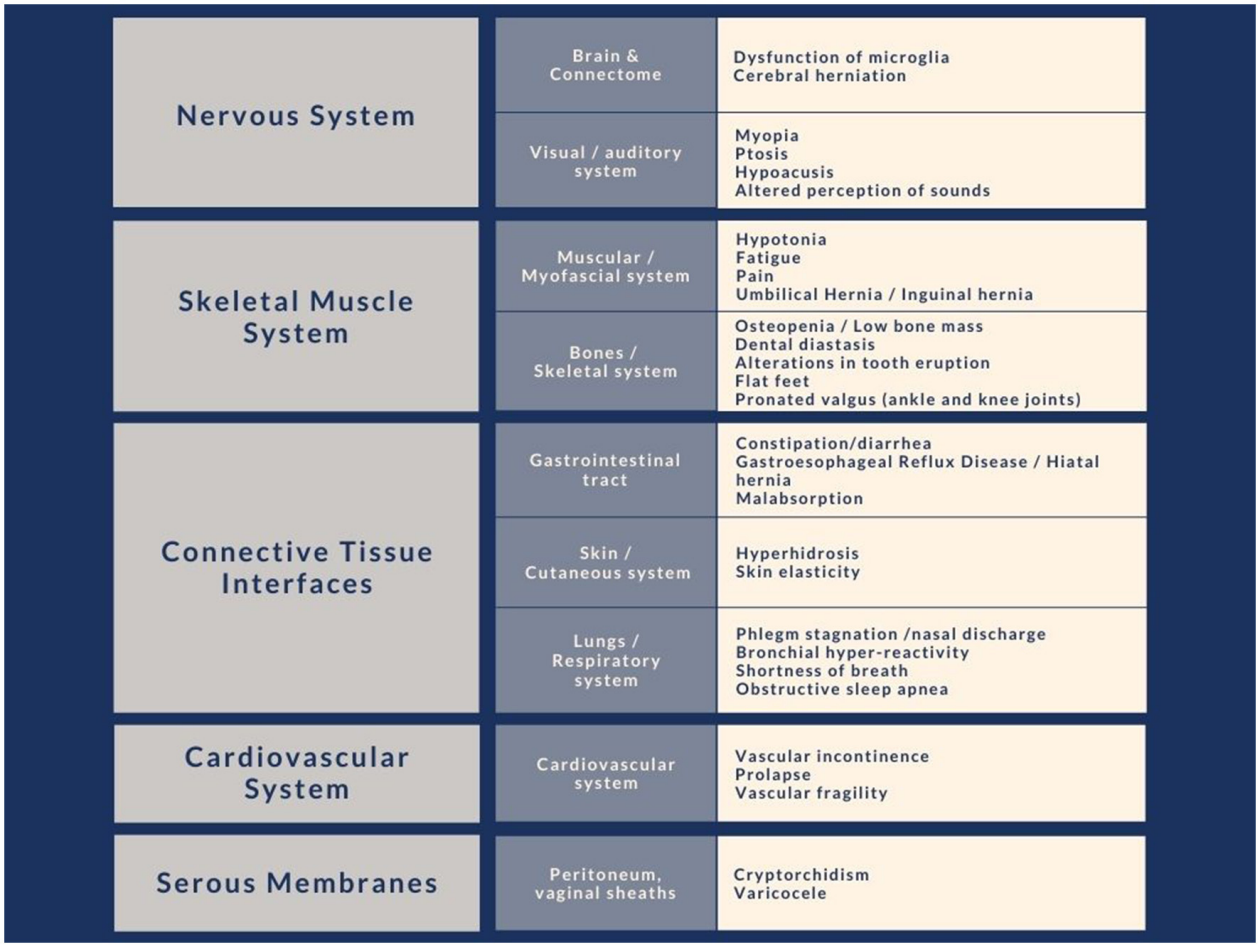
Clinical signs due to alterations of the connective system commonly found in subjects with autism, divided by body site. We have noted in our clinical experience that these characteristics have a particular recurrence within the immediate family.

Connective tissue constitutes the interface that allows a functional separation between internal and external environments with an interchange function between the two. The anatomical sites that play an interface role with the external environment are, in particular, gut, skin, and pulmonary alveoli (Figure 2).

#### Gut

People with ASD often suffer from intestinal dysfunctions whose relevance is recognized in the literature (33). Connective tissue is highly represented in the gut, where it plays an important role in regulating intestinal permeability and related absorption functions, in favoring or altering the number of microbial populations and in allowing peristaltic movements. Alterations in intestinal connective tissue result in increased immunological reactivity due to increased permeability, which promotes the absorption of pathogens present in the intestinal lumen; this anomalous reactivity has repercussions in turn on the cognitive sphere and on socio-linguistic abilities (34–36).

The intestinal barrier consists of enterocytes joined to each other by “tight junctions,” connection structures that regulate the degree of epithelial cells cohesion and, consequently, the passage of proteins and pro-inflammatory substances through the paracellular spaces. Several pathological conditions concerning intestinal mucosa are highly prevalent in ASD, from irritable bowel syndrome to inflammatory bowel diseases (37). There are also conditions of particular visceral laxity that allow easier passage of toxic compounds and refined sugars that cause damage to the epithelial cells and which, consequently, are found in the urine (33, 38). Areas of damage were found particularly in the colon, but also in the stomach, after biopsies performed during endoscopies (39–41). Food intolerance to gluten, lactose, casein, glucose, and mannitol is a frequent occurence in ASD, and intestinal and skin function alterations are often found (37). The presence of antigens on the epithelium surface of the intestinal lumen, as in the case of gluten in the proximal or distal gut, can also lead to alterations in glandular secretion at the epithelial barrier level, which can result in a further increase in zonulin or claudin synthesis (37). Also, in this case, alterations in permeability may occur which favor the entry of infectious agents into the bloodstream, resulting in immune reactivity against some of them.

Different bacterial populations coexist on the surface of intestinal mucosa with lymphocyte cells, which represent the guardians of immunity and prevent the development of pathologies (41). Intestinal microbiome includes highly variable bacterial colonies, which in ASD are greatly affected by dietary intake (folate, vitamin B12, etc.), inflammation mediators, oxidative stress and the secretion levels of the various hormones that regulate gastrointestinal tract motility (42). Anaerobic bacteria are particularly present in the large intestine, especially Bacteroides and Lactobacilli. These microorganisms are associated with the fermentation of polysaccharides taken with the diet (43). In case of an alteration in the intestinal barrier, elements of the external environment can support processes that mediate the onset of inflammatory conditions. In this regard, an often-reported example is bacterial overgrowth that leads to a reduced concentration of Gram-negative such as Bifidobacteria and to the relative growth of Clostridia, which can be found in stool analysis and that represent a clinical sign of tissue inflammation (44).

#### Skin

Skin, which covers the entire body, plays a shaping role thanks to its elastic characteristics. Similar to the intestine, skin has regulation functions, protects from harmful noxae, and plays protective and thermal insulation roles. The skin also contains receptors that transmit sensory signals. In ASD, immune-mediated inflammatory pathologies of the skin can occur with a 5:1 frequency compared to the healthy population; and this immune-mediated dysregulation predisposes the body to inflammation and alterations in sensory perception and thermoregulation (45), observable in both the more superficial and deeper layers of the skin, dermis, and hypodermis.

The presence or absence of bacterial colonies on the skin surface makes the skin enclosure “barrier” more or less effective. As a demonstration of this, a study by Slingsby et al. highlighted the presence of skin lesions in children with ASD. Their study population was more likely to have various forms of dermatological alterations, especially on the legs, knees and forearms (46). However, these cases of skin lesions must be thoroughly investigated to exclude self-injurious behavior.

Another study by Yao et al. showed how the activation of platelet and vascular endothelium could contribute to the development and clinical manifestation of the aforementioned dermatological alterations in autism. More precisely, they described how an altered function in platelet aggregation can lead to greater cutaneous dysfunctions, with dermatological lesions attributable to capillary dysfunction due to oxidative stress (47). The basis of immune-mediated inflammation is an increased nitric oxide level as well as an altered ratio between CD4 and CD8 lymphocytes (48). As in other childhood syndromes of known etiology, melanocyte alterations such as vitiligo were also found. This could lead to reduction of pigment that protects the skin from the sun's rays. Consequently, exposure to the sun can lead to erythematous reactions (45). Skin alterations on atopic bases and mediated by increased plasma immunoglobulin E levels were also described in the study (49). Skin allergy manifestations are known as dermatitis or urticarial conditions. On the immune-mediated side, particularly in atopic etiopathology, the onset of these disorders is promoted by predisposing genetic factors activated by environmental exposures to specific agents such as particular food substances (49–52). In our clinical experience, for example, eczema is one of the most frequently encountered atopic diseases in ASD, with a prevalence reported in the literature of up to 10.2% (52).

There are different types of tactile receptors in the skin, each specific to the characteristics of the stimulus. The main ones are thermal (thermoreceptors), pain (nociceptors), pressure, and vibratory (mechanoreceptors) receptors. These structures send information to the thalamus which, after sorting and synchronization, sends it in turn to the sensorimotor cortex (53). With dermoscopic analyses, it was also observed that ASD subjects had atypical skin structural characteristics—for example an increased presence of skin fissures or red streaks (54). Sensory transmission delay can also be found in about 69% of cutaneous and subcutaneous alterations. This factor correlates with alterations in the sensory processing of touch (55). Altered synchronization may occur at the thalamus or at the cortex. If the alteration is at the thalamus, an ASD subject could perceive the characteristics of the object but would find it difficult to integrate them. At the cortex, the signal can arrive with altered intensity and can be associated with hypo- or hyper-sensitivity; consequently, the response of the ASD subject to environmental stimuli will also be altered (56, 57).

Skin also protects the body from thermal changes and maintains the internal temperature constant. Increased sweating has been observed in children with ASD, and one is likely to notice a greater presence of sweaty skin especially on the extremities (hands and feet). These atypicalities may be linked to dysfunctions of the sensory receptors present in the different skin layers, to alterations in the degree of skin tissue nutrition or to impaired hormonal regulation of the trophism and blood circulation in the skin and subcutis (54). This also explains the frequent findings of thinning of the skin, excoriations, and skin striae in ASD subjects. Moreover, there is an inadequate release of mediators in their skin and subcutaneous layers that counteract inflammation; this predisposes the skin to pro-inflammatory events (54). As already reported in regard to intestinal mucosa, bacterial colonies that perform an immunological surveillance function are also particularly present in the skin layer, not only helping to keep the defenses active against pathogenic events, but at the same time influencing secretion by the sweat and sebaceous glands (54).

#### Lungs/Capillary Alveolus

A third connective interface is the “alveolo-capillary barrier,” whose dysfunctions have sometimes been found in ASD. Pulmonary connective tissue, in addition to having a supporting function, is characterized by greater elasticity, a peculiar characteristic that allows the lung to contract and expand in the two breathing phases. In addition, the connective tissue, thanks to its structure organized in alveolar branches, allows the exchange between the substances contained in the air and blood. Alveolar capillary dysfunctions could be influenced by immune-mediated factors, atopic processes, and inflammatory causes, of which a triggering pathogen may or may not be recognized (58, 59). The most frequent clinical manifestations can include allergic asthma, bronchial hyper-reactivity, or inflammatory pathologies of the pulmonary interstitium, supported, at least in some cases, by high levels of pro-inflammatory metabolites like cytokines or short-chain fatty acids (60). A 2008 study carried out on a sample of ASD subjects in Egypt demonstrated the correlation between autism severity and respiratory manifestations like exhalation wheezing, sense of chest constriction, and nocturnal breathing difficulty unresponsive to common bronchodilators (60). This study also reported a correlation between itching, nasal discharge and sense of nasal obstruction following exposure to environmental allergens. In a 2009 Swedish study by Larsonn et al., a greater prevalence of asthma was found in children with ASD between 4 and 8 years of age (61). In other words, the presence of heterogeneous respiratory conditions that share breathing difficulties and increased mucus production is often reported in ASD patients. The accumulation of secretion in the upper respiratory tract can also depend on a lack of coordination of the vibrating lashes. Compared to healthy subjects, asthma, and allergic rhinitis have a 5:1 prevalence in ASD, and food-related factors such as cross-allergies and food sensitivities can particularly affect both manifestations (45). The co-occurrence of skin and respiratory allergies and of various cross-allergies is observed in many children with autism. Some studies also report altered local permeability of the bronchial mucosa that, as previously mentioned regarding the intestinal mucosa, appears to facilitate inflammatory processes (60). Secretion stagnation causes an increase in density and leads to the proliferation of bacterial populations and pathogenic germs. These phenomena can account for the increased incidence of viral and bacterial infections in children with ASD, especially in autumn and winter. Furthermore, during the recent global pandemic, an increased susceptibility to SARS COV-2 infection and the development of more severe forms of the disease have been observed in autistic children (62).

Finally, it is possible to detect nocturnal asthmatic forms or recurrent coughing, caused by gastro-esophageal reflux, correlated to a ligamentous laxity of the cardias. This phenomenon occurs mostly at night when the child is lying down, due to the increased secretion of acid within the gastric lumen during the digestive phase (63, 64) which can damage the respiratory tract, especially in children with more severe forms of ASD.

#### Cardiovascular System

Components of mesodermal origin also predominate in the cardiovascular system, where they play an important role in promoting the distribution of oxygen, nutrients, and hormones throughout the body and allow the immune system to perform its function in all districts. Recent studies have documented that insufficient immune defense in autism leads to impaired antioxidant capacity and subsequent increased production of free radicals (65). In order to maintain adequate pressure control and capillary flow regulation, the body requires adequate synchronization between the parasympathetic and sympathetic systems, the two components of the autonomic nervous system. When instead of working in synergy they are partially mutually in contrast with each other, the alteration of their reciprocal synchrony can lead to minimal alterations within the cardiovascular system (66, 67). In other words, in presence of capillaries with greater resistance to blood perfusion, the heart needs to raise the blood pressure level. Peripheral resistance can thereby be influenced by vessel wall elasticity (68, 69). In this regard, the ability to regulate the diameter of small vessels is influenced by the arterial stiffness index or by the reduced elasticity of blood vessel walls, which also affects the arteries' ability to impart a pulsatile force to the bloodstream (70).

The more relevant these alterations, the more the body is exposed to stress, such as during physical activity when the demand for peripheral perfusion is greater due to the organs' increased metabolic requirements. Regarding the altered blood pressure of children with ASD, in addition to the regulation of vessel volume, changes in electrocardiogram heart rate tracings (heart rate variability) have also been found and appear to be related to anxiety disorders—intrusive and obsessive thoughts affecting daily activities (71). A 2016 Japanese study also confirmed that ASD children with reduced vagal activity at rest, measured by precise parameters of electrocardiographic tracing, had alterations in self-regulation skills and behavioral dysregulation abilities associated with unpredictable and unusual visual-auditory stimuli (72). A 2013 American study found that dysfunctions in cardiac autonomic control are directly related to dysfunctions in the attentional, emotional, and/or cognitive spheres and, consequently, in social interactions with peers. This is even more relevant for children with neurodevelopmental disorders, particularly those with attention deficit/hyperactivity disorder (ADHD) or other disorders presenting hyperactivity and inattention traits (73).

#### Visual System

The visual system is among the sensory channels which more contribute to the perception of one's body position in space and in relation to others (74). Connective tissue represents the main component of the corneo-scleral scaffold and acts as a diopter and a support structure for the vascular (uvea) and nerve (retina) tunics. The eye is also protected in its orbit by connective tissue, which is also part of the oculo-extrinsic muscle tendons (75). The ocular movement is therefore harmonized by the connective pulley system (76). In the retina, on the other hand, microglia, astrocytes and Müller cells help to maintain homeostasis and optic nerve organization, particularly in the head (pre-laminar, laminar, and retrolaminar portion). In fact, the order of nerve fibers and their arrangement are crucial for the development of visual pathways (77). The visual system develops early in the developmental age to enable perception of the surrounding environment; a remarkable frequency of visual disturbances and alterations in conjugated ocular motility has been observed in subjects with ASD (75). Asymmetry of the saccades and difficulty in slow tracking movements of moving targets have been reported. These alterations may indicate impaired functional integrity of the brain networks in ASD in developmental age and may help in understanding attention deficits (78). The eyes converge on the object of interest if activities of the extrinsic ocular muscles are synchronized. In autism a deficit in coordinated motility, in both rapid pursuit and convergence, is observable. This can lead to strabismus, which may require medical or even surgical correction. Other observable deficits, as already mentioned, may relate to visual acuity. For example, at birth or in early childhood, it is not uncommon to find myopia or astigmatism; other ophtalmologic disorders such as strabismus or amblyopia are also common (79). These impairments can have repercussions of favoring the appearance of stereotyped behaviors in response to difficulties in perceptual interaction of space. Another component of the visual system that can be altered in autism is the ability of the sphincter and pupillary dilator muscles to accommodate the pupillary reaction to ambient light (photomotor reflex). As regards the retinal structure, aspects of hyper-reactivity to environmental stimuli like lights, color contrasts, or associations of geometric shapes have been reported in subjects with ASD in atypical and unexpected behaviors, similar to what happens for other sense organs that present phenomena of hyper-sensoriality (80, 81). In fact, the GABAergic and glutamatergic circuits are altered in ASD subjects, with consequences on the excitation-inhibition system and this could be the reason for hypersensitivity to stimuli. Analysis of retinal function using full-field electroretinogram of ASD subjects has shown alterations in both cones and rods, which are then partially compensated at the post-receptorial level (82, 83). The cortical circuits responsible for the transmission of visual stimuli also have an anomalous development in subjects with ASD. In fact, microglia is responsible for pruning of the synapses, which contributes to the connectivity and refinement of the visual circuit. In particular, pruning of retino-geniculate synapses by microglia in the axonal innervation of the lateral geniculate nucleus is essential for the correct integration of perceived visual stimuli. Just as occurs, for example, in the deprivation/restoration of vision, the visual experience itself modulates the visual circuits through the microglia that guide the formation of synaptic fissures and interposition of astrocytes fissures and the interposition of astrocytes (76). Finally, sectorial retinal thickening and increased vascular density have been found in subjects suffering from ASD, without an increase in blood flow (84). These findings suggest that patients with ASD have impaired self-regulation of blood flow in relation to metabolic demands.

It is therefore natural to ask whether the atypical processing of the social stimulus at a visual level found in people with ASD may be due to the altered synchronization of the signals emitted by the retinal receptors or to the incorrect interpretation of the signal itself by the visual cortex.

#### Auditory System

In our experience, bizarre reactions to auditory stimuli can also be found in subjects with ASD and therefore early audiometric examinations are recommended. Already in 1983 Gillberg et al. reported a hearing loss in 13.3% of their autistic population (80). This was attributed to a higher prevalence of middle ear infections (e.g., recurrent otitis media), which could be justified mainly by two aspects: reduced efficiency of the immune system and a particular anatomical conformation of the eardrum (81). The outer, middle, and inner ear are composed mainly of connective tissues. The latter is involved in various tasks, both structurally and in conducting sound (bone or air). With these facts in mind, clinicians should consider both conduction routes when performing audiometric tests.

Latency anomalies have been found in groups of children with ASD, in particular in recordings made at the brainstem, the reason being attributed to the maturation of the myelin sheath (85, 86). Cortical auditory evoked potentials also show alterations in subjects with ASD at the level of auditory integration in the cortical site (87). Alterations of the audio-perceptive system can lead to dysfunctions of overt and covert attention, which are the most conscious levels of attention (such as selective attention) as well as the most unconscious (more on a perceptual level of environmental stimuli) (88). A further study reports significant asymmetry between the two hemispheres in the reception and processing of stimuli (89). In relation to specific behavioral patterns of avoidance or search for certain auditory stimuli, typical of the clinical ASD phenotype, neuroimaging studies have reported increased involvement of the limbic system, compared to the norm (90). The limbic system is particularly important for the attribution of emotional and gratification connotations, thus justifying peculiar auditory patterns.

From the clinical point of view, recent studies report signs and symptoms of a high hearing threshold of environmental stimuli in ASD subjects; for instance, these subjects may have a reduced perception of sounds (91). Hearing difficulties could be due to difficulty in discriminating and recognizing sounds, especially those of a social nature, with respect to non-significant auditory stimuli (92). Other authors relate hearing loss to an attention deficit shifting toward significant auditory stimuli. In fact, the lack of sociality can be accentuated by an auditory deficit that interferes with the perception or decoding of stimuli (91). In addition, altered modulation of sensory processing in childhood reduces a child's ability to carry out daily autonomic activities, especially those involving communication (93).

## Genetics and Epigenetics

Connective tissue fulfills a fundamental role as the interface between external and internal environments. It is therefore important to describe how the relationship between connective tissue and environment is mediated by genetic and epigenetic factors in ASD subjects. In contrast to other neurodevelopmental disorders with polygenic contribution, the phenotype in autism is expressed by multiple alterations at several genetic loci with variable expressiveness. In recent years autism has frequently been thought to result from a combination of genetic susceptibility and environmental triggers. Environments to which the child is exposed during growth influence the susceptibility of those gene alterations traditionally related to ASD (94). Much has been studied about the genetics of autism and some of the genes involved in the spectrum are responsible for functions and structures typical of connective tissue. There are particular conditions such as ASD or Rett syndrome, previously cataloged within the DSM-IV-Text Revision in pervasive developmental disorders, which are characterized by cognitive alterations (social skills) and alterations in connective tissue, with hyper-extensibility of tendon structures (95). The conditions in which ASD and connective tissue alterations are associated can be determined by several autosomic dominant (AD), autosomic recessive (AR) or X-linked mutations. These mutations have been found in both autism and the condition of joint hyper-mobility. Regarding AD mutations, when the CDK8 gene is altered, it involves anomalies of the intellectual developmental disorder with hypotonia and behavioral abnormalities. AR mutations include the NTNG2 gene which supports neurodevelopmental disorders with behavioral abnormalities, absent speech, and hypotonia, whereas one gene that relates to the X-linked mutation is FMR1, which relates to Fragile X syndrome (95).

The gene in the 15q11-q13 position is one of the most interesting genetic loci frequently associated with developmental age disorders and which involves alterations in psychomotor, somatic, behavioral and emotional development trajectories. Thirty characteristic genes in this arm could be associated with the prevalence of ASD. A particular gene, UBE3A, encodes the same development trajectory for modulating proteins. The same happens for genetic pathologies in childhood such as Prader-Willi syndrome (96).

## The “Connectivome Theory”

Based on the considerations reported in the previous paragraphs and in particular reference to the relationship between connective tissue and the different organs and tissues, we propose herein a new multidimensional perspective that offers a methodological clinical approach to get a more global view of the ASD phenotype. Considering only neurofunctional factors might be a limited way of investigating ASD. Today, scientific research is providing new information that gives us an increasingly global view of the interaction between psyche and soma. If, in some contexts, the Cartesian separation between psyche and soma is still applicable, in the context of neurodevelopmental disorders this is no longer appropriate. Therefore, a more thorough study of the physical sphere can be an important starting point to better understand the disorder itself. In our discussion we have explained that, besides support and adhesiveness in the various body sites (Figure 2), the connective tissue also performs other functions, such as connection, regulation, and modulation between cellular elements belonging to different tissues, particularly in the CNS through the activity of microglia, which shares its mesodermal origin.

Connectivity dysfunctions can have important neurofunctional connotations. In this regard, ASDs represent interesting and distinct disorders. They include, on the one hand, altered central connectivity and, on the other, somatic alterations at multiple body levels. In body sites where connective tissue plays a salient function, a greater co-occurrence of multiple and multi-level somatic alterations, with greater or lesser severity, may be found. However, autism may not be the only disorder to benefit from the use of this interpretative model. In fact, the same characteristics found in the autism spectrum can be found in other neurodevelopmental disorders, particularly in those conditions often in comorbidity with it (ADHD, Tourette syndrome).

Referring to the aforementioned concepts, we deemed it necessary to coin a new term since in our opinion there is no definition in the literature that encompasses all the connective structures of the human body in the same way that the term “connectome” includes the whole of neural connections. The word “connectivome,” therefore, seemed the most natural choice to include the central and “psychic” concept of the “connectome” also with the peripheral and “somatic” level, implying the close correlation between the two systems.

**Figure 3:**
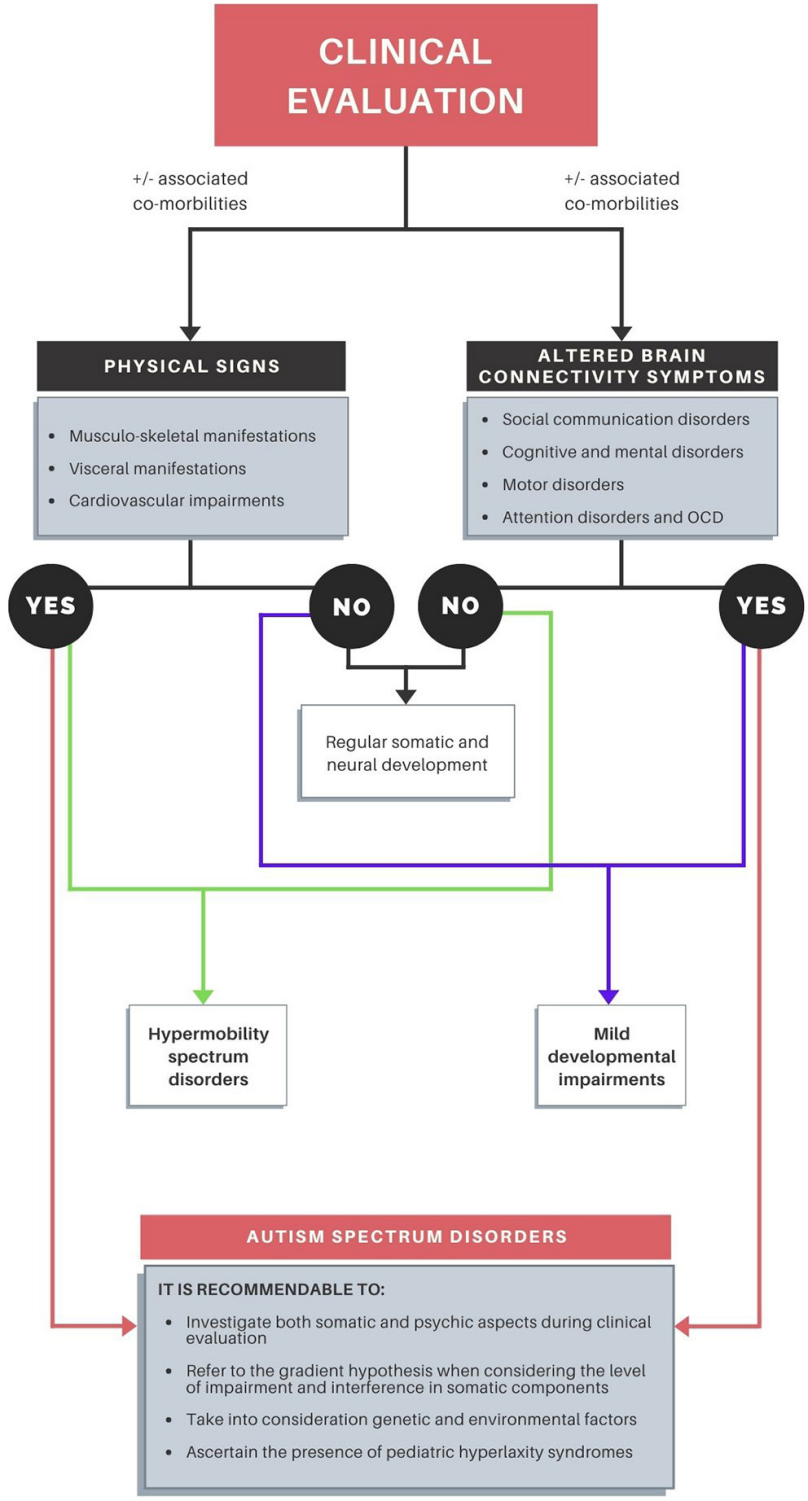
Clinical evaluation model according to the “connectivome theory”.

In the following points we present the considerations that constitute the “connectivome theory” (Figure 3).

1. The clinical evaluation of a person with ASD cannot be separate from a somatic examination; it must be transversal and allow for differentiation of somatic forms with mono-district involvement, forms with segmental involvement and forms with multiple or generalized involvement. It is also important to note that not all clinical signs of ASD simultaneously manifest at birth or at the onset of the disorder, but could arise at different stages of life.
2. Clinical findings should be interpreted in light of a gradient hypothesis, according to which tissue alterations of various degrees can be found depending on ASD severity. In our direct clinical experience, we have observed that higher levels of ASD severity correspond to more significant somatic alterations, in particular at body sites with greater connective tissue contribution. It would be helpful if future studies investigated the repercussions of somatic alterations in the connective tissue on the functional profile.
3. Connective interfaces (particularly skin, alveolar-capillary barrier and intestinal mucosa) represent privileged sites of exchange between internal and external environments. Having established that autism is a representative condition in which genetic and environmental factors are preponderant, the connectivome theory allows a wider interpretation of the epigenetic role in ASD.
4. In the previous paragraphs we considered the somatic characteristics in the child, but the clinician should not neglect elements of a more anamnestic nature, such as the chronic psycho-physical fatigue trait, commonly found in HSD. Fatigue can manifest itself physically or mentally and can significantly affect daily life. Physical fatigue is mostly reported in peripheral districts (muscles, intestines, etc.) and can also affect the immune system on the genesis of immune-mediated conditions. Mental fatigue, on the other hand, manifests as attention difficulties or intrusive thoughts, which in turn can be expressed through anxiety and mood symptoms. A clinical proposal that is progressively affirming itself associates chronic somatic fatigue, where specific body composition analysis tools detect pictures of altered connectivity and ligament laxity, with the psychopathological manifestations described above. In fact, the latter tend to appear within hyperlaxity syndromes of the pediatric age, characterized by muscular hypotonia and joint hypermobility.

The connectome model, although still current and whose validity has been confirmed by studies that found similar evidence in terms of altered connectivity, could be considered “encephalocentric” (97, 98). On the contrary, according to the “connectivome theory,” elements of a somatic nature that were not adequately valued in the connectome model now take on an equally important role, without the hierarchization of soma and psyche.

## Data Availability Statement

The original contributions presented in the study are included in the article/supplementary material, further inquiries can be directed to the corresponding author/s.

## Author Contributions

LZ, MC, and NZ contributed to conception and design of the study. LG and MC organized the first draft of the manuscript and the first version of the manuscript. NZ and GG performed the revision of the manuscript. GG performed the English translation. All authors contributed to manuscript revision, read, and approved the submitted version.

## Conflict of Interest

The authors declare that the research was conducted in the absence of any commercial or financial relationships that could be construed as a potential conflict of interest.

## Publisher's Note

All claims expressed in this article are solely those of the authors and do not necessarily represent those of their affiliated organizations, or those of the publisher, the editors and the reviewers. Any product that may be evaluated in this article, or claim that may be made by its manufacturer, is not guaranteed or endorsed by the publisher.
